# Splenic Infarct: A Rare Complication of Infectious Mononucleosis in a Monospot-Negative Patient

**DOI:** 10.7759/cureus.76127

**Published:** 2024-12-21

**Authors:** Arshia Ahmed, Salman J Khan, Sara Tariq, Lela Adeoshun

**Affiliations:** 1 Internal Medicine, Guthrie Lourdes Hospital, Binghamton, USA; 2 Public Health, University of Massachusetts Amherst, Amherst, USA

**Keywords:** abdominal pain, ebv, infectious mononucleosis (im), monospot, splenic infarcts

## Abstract

Splenic infarction with infectious mononucleosis (IM) caused by Epstein-Barr Virus (EBV) has been reported as a rare complication of IM. The monospot test, often used to diagnose EBV-related IM, may produce false-negative results, especially in atypical presentations or early stages of infection. This report describes the case of a monospot-negative patient who developed splenic infarction as a complication of IM. The pathophysiology of splenic infarction in IM remains poorly understood, though it is thought to be related to splenic congestion, thrombophilia, or the direct impact of EBV on the spleen's vasculature. This case report illustrates the diagnostic challenges and clinical significance of splenic infarction in a 21-year-old female who presented with fever, jaundice, fatigue, and mild abdominal discomfort. She was found to have splenic infarcts on imaging. Her monospot test was negative. However, she was diagnosed with EBV infection on EBV viral capsid antigen (VCA) antibody testing. This case also emphasizes the importance of clinical vigilance in diagnosing and managing rare complications of IM, even in the absence of positive monospot results, and highlights the need for further investigation into the mechanisms that predispose certain individuals to splenic infarction during infectious mononucleosis.

## Introduction

Epstein-Barr Virus (EBV) is a common virus belonging to the *Herpesviridae* family and is known for causing infectious mononucleosis (IM). IM is characterized by tonsillar pharyngitis, fever, and posterior cervical lymphadenopathy, most commonly affecting individuals aged 5 to 25 [1]. The monospot test is the initial test to detect heterophile antibodies of IM. Some patients with IM have negative monospot tests. In these patients, testing for antibodies to viral capsid antigens is recommended [2]. Patients with IM mostly recover in a few weeks, rarely developing any complications [3]. The minority of the IM patient population develop neurological, hematological, cardiac, and gastrointestinal complications which are less than 1% [3,4]. Complications affecting the spleen encompass splenomegaly, splenic rupture, and, less commonly, splenic infarction [4]. 

Splenic infarction is a relatively rare complication, and patients usually present with acute abdominal pain, splenomegaly, and other clinical features indicative of splenic dysfunction. In this case report, we are reporting a rare complication of IM in a 21-year-old female who had a negative monospot test but positive EBV viral capsid antigen (VCA) antibody testing and was found to have splenic infarct on imaging. 

## Case presentation

A 21-year-old female with no previous medical history presented to the emergency department (ED) with complaints of high-grade fever along with generalized weakness for 10 days and yellowish discoloration of the sclera for five days. A week before the ED presentation, the patient had a walk-in visit and was prescribed cefdinir because of a positive streptococcus a infection. The patient’s fever did not subside after the antibiotic. She was prescribed another antibiotic, azithromycin, for another three days. The patient continued to have a fever for almost 10 days and was presented to the ED. The patient did not have acute abdominal pain; however, she complained of dull, aching, non-radiating pain in the epigastric and left hypochondriac region of the abdomen of grade 4/10 in intensity, which was not associated with vomiting or diarrhea. There was no history of sick contacts, tick bites, animal exposure, or recent travel. The patient denied ingestion of exotic fruits or mushrooms. The patient vapes intermittently and takes oral contraceptive pills (OCPs) for two years. The patient’s vitals were stable, and the physical examination was unremarkable except for jaundice and a palpable liver edge. There was no lymphadenopathy, hepatic flap, rash on the body, dental abscess, or caries. 

Her labs on admission revealed a normal white blood cell (WBC) count at 7.2 x 10^9^/liter and low hemoglobin and platelets at 11.5 grams/deciliter (g/dL) and 188 x 10^9^/liter, respectively. The peripheral smear showed mild absolute lymphocytosis with a lymphocyte count of 51,000 cells/microliter (mL) with atypia and a neutrophil count of 31,000 cells/mL. Her rapid group A streptococcus test was negative. She had elevated liver function tests with aspartate aminotransferase (AST) 271 units/liter (U/L), alanine aminotransferase (ALT) 368 U/L, total bilirubin 5.0 milligrams/deciliter (mg/dL), and alkaline phosphatase (ALP) 392 U/L. Her monospot test was negative. Labs on the day of admission are shown in Table 1.

**Table 1:** Labs on the day of admission WBC: White Blood Cell; AST: Aspartate Aminotransferase; ALT: Alanine Aminotransferase; ALP: Alkaline Phosphatase.

	Labs on the day of admission	Normal Range
WBC count (k/ul)	7.2	4.5-11.0
Hemoglobin (g/dl)	11.5	12.0-15.5
Platelet count (k/mcL)	188	150-400
Lymphocyte count (cells/mL)	51000	1000-4800
Neutrophil count (cells/mL)	31000	2500-7000
AST (U/L)	271	8-48
ALT (U/L)	368	0-30
ALP (U/L)	392	44-147
Total bilirubin (mg/dl)	5.0	0.2-1.2
d-dimer (ng/ml)	110	220-500
Fibrinogen (mg/dl)	112	200-400

**Figure 1:**
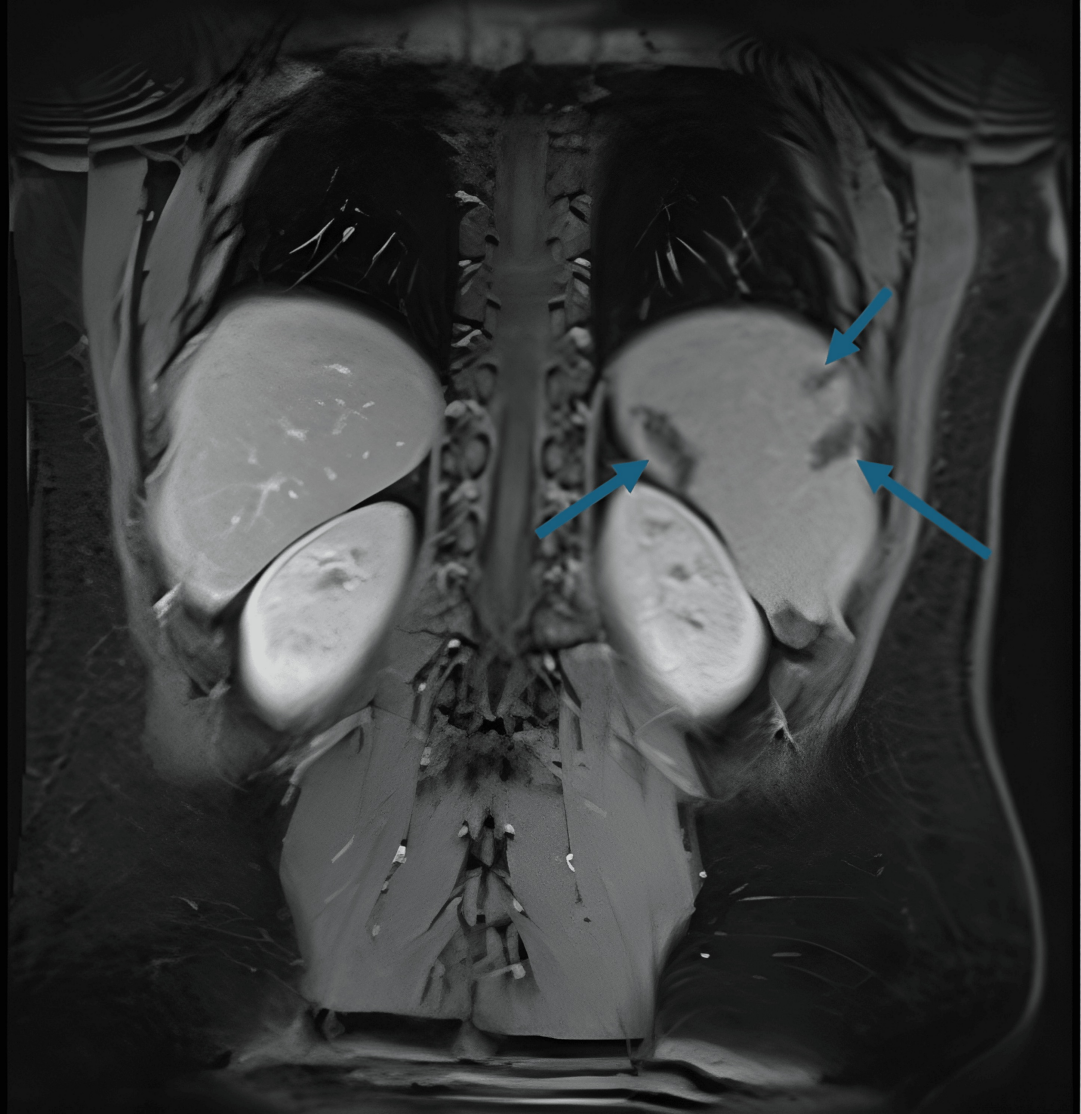
MRI T1-weighted fat-suppressed coronal image showing numerous scattered areas of wedge-shaped hypointense signals consistent with splenic infarcts (blue arrows)

**Figure 2:**
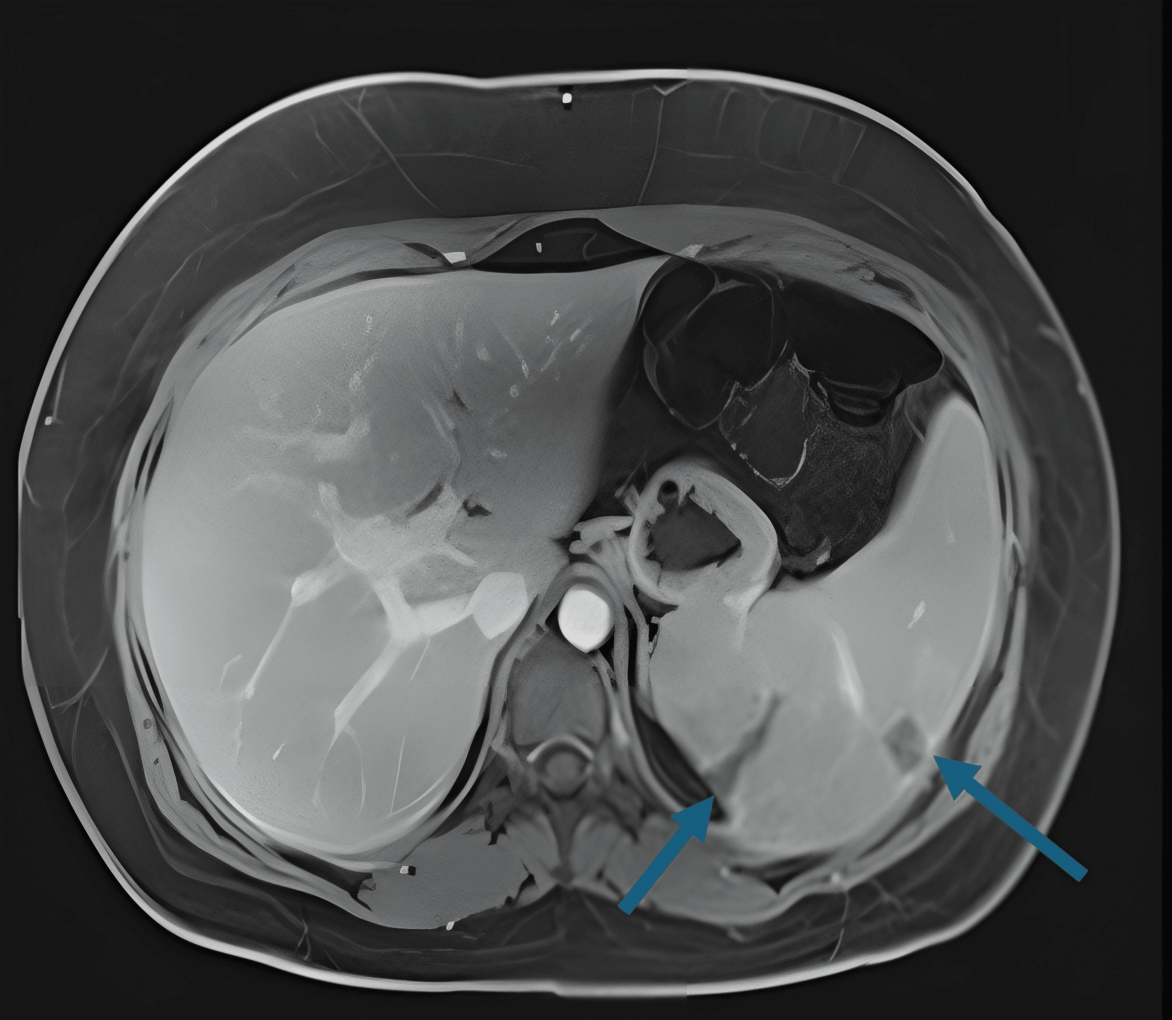
MRI T1-weighted fat-suppressed axial image showing numerous scattered areas of wedge-shaped hypointense signals consistent with splenic infarcts (blue arrows)

The patient’s CT chest/abdomen/pelvis without contrast revealed mild splenomegaly along with multiple wedge-shaped peripheral foci of hyperattenuation within the spleen, likely representing infarctions and mild hepatomegaly. Magnetic Resonance Imaging (MRI) of the abdomen, without contrast, showed numerous scattered areas of wedge-shaped hypointense signals consistent with splenic infarcts (Figures 1, 2). 

Differential diagnoses for this patient included acute hepatitis, autoimmune disease, viral illness, thromboembolism, and inherited coagulopathy, given young age and acute elevation of hepatic enzymes. Protein S and C were within normal limits. Lupus anticoagulant, anticardiolipin immunoglobulin M (IgM), and antinuclear antibody (ANA) tests were negative. She had negative human immunodeficiency virus (HIV) and cytomegalovirus (CMV) tests. Her hepatitis panel was negative. Based on clinical suspicion, the decision was made to do EBV VCA antibody testing, which confirmed EBV infection, hence IM. The diagnosis of splenic infarction secondary to EBV infection was made based on clinical presentation, laboratory findings, and imaging results. 

**Figure 3:**
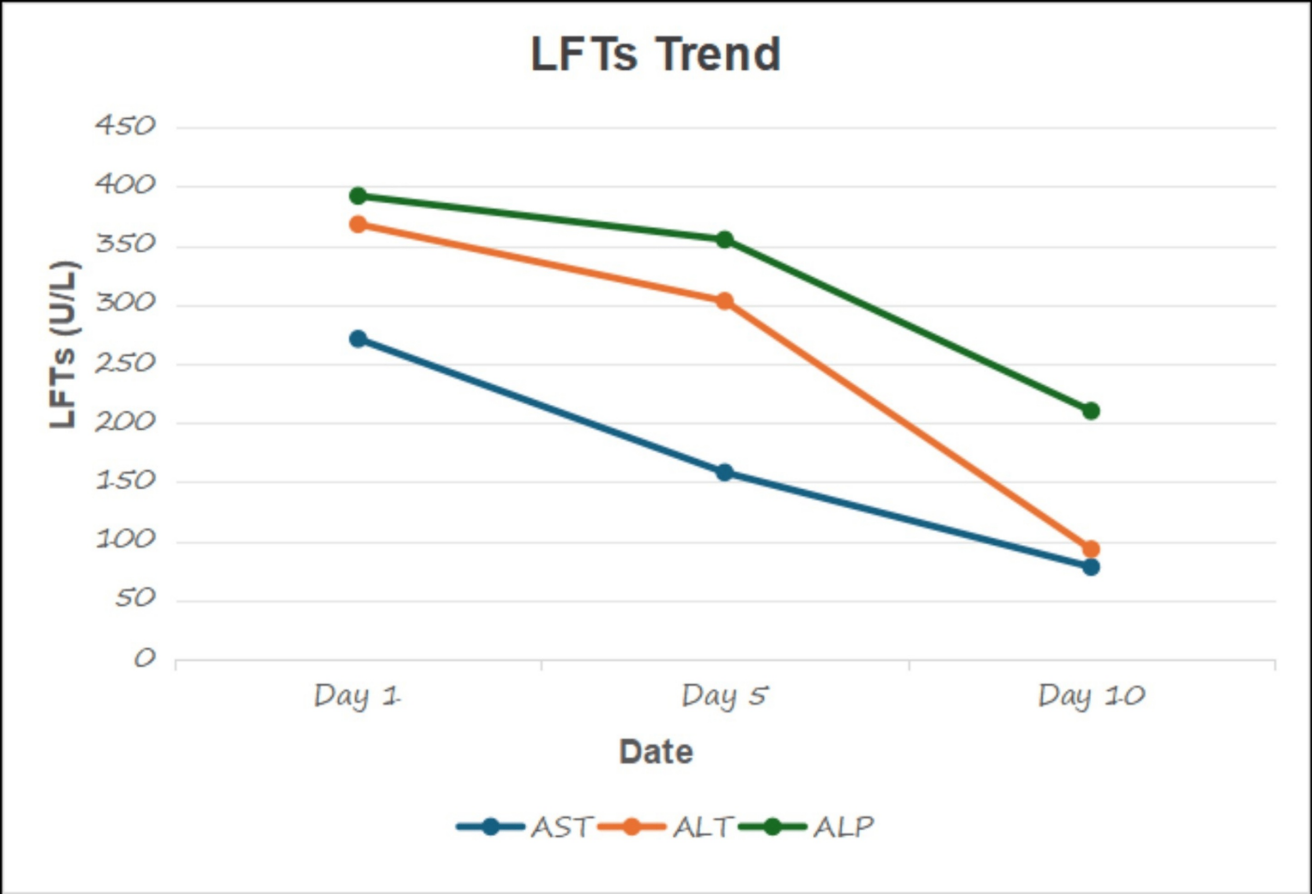
LFTs trend LFTs: Liver Function Tests; AST: Aspartate Aminotransferase; ALT: Alanine Aminotransferase; ALP: Alkaline Phosphatase; U/L: Units/liter.

The patient was managed conservatively with supportive care, including hydration and pain management. Her liver function tests (LFTs) started to trend down and her symptoms improved (Figure 3). She was discharged after 14 days. She was advised to avoid physical exertion and contact sports to prevent splenic rupture. Regular follow-up was arranged to monitor the resolution of symptoms and assess spleen function. Her symptoms of fatigue and fever improved in a couple of weeks, and her LFTs started normalizing with a resolution of jaundice. 

## Discussion

Patients with IM usually present with sore throat, fatigue, fever, and cervical lymph node enlargement. In a minority of patients, it progresses to neurological, hematological, cardiac, and gastrointestinal complications [3-5]. Of all these complications, splenic infarction is an uncommon but serious complication of IM that developed in our patient. Splenic infarction in IM is likely multifactorial, stemming from EBV-induced hypercoagulability, transient lymphocytic infiltration of the splenic vasculature, and the reactive splenomegaly compressing the splenic arteries, leading to reduced blood flow and infarction [5-9].

Yan Li et al. described three cases of splenic infarction attributed to infectious mononucleosis; all of these cases had abdominal tenderness in the left upper quadrant and fever [10]. One case had submandibular lymphadenopathy. CT imaging showed splenomegaly and multiple wedge-shaped hypodensities throughout the spleen consistent with infarcts [10]. Our patient did not have lymphadenopathy or any tenderness in the abdomen. However, she had a fever and sore throat with mild abdominal discomfort and was found to have multiple splenic infarcts. This observation shows that patients can have minimal abdominal symptoms and yet can have splenic infarctions. 

Splenic infarction can present with a wide range of symptoms, including minimal abdominal pain to life-threatening hemorrhage or sometimes with no symptoms at all. CT scan is the most appropriate choice of imaging if splenic infarction is suspected, as ultrasound sensitivity is only 18% [11]. Although infectious mononucleosis typically manifests with fever, lymphadenopathy, and pharyngitis, splenic complications such as infarction and rupture, while rare, can lead to severe morbidity and should not be overlooked. While the majority of IM infections are self-limiting, complications like splenic infarcts warrant prompt recognition and management [12]. 

In our case, the diagnosis of IM was initially delayed due to the patient’s atypical presentation of mild abdominal discomfort and negative monospot test. The persistence of fever, jaundice, and elevated liver enzymes prompted further investigation. Imaging revealed splenic infarction, a finding that, although uncommon, has been reported in association with EBV (Figures 1, 2). 

The importance of a thorough evaluation in patients with prolonged fever and unexplained symptoms cannot be overstated. While infectious mononucleosis is often diagnosed clinically or via a monospot test, its limitations in sensitivity, especially in early infection or older patients, underscore the necessity for specific EBV serologies, as demonstrated in this case [11,12]. Early identification of splenic involvement through imaging is vital, as infarction may progress to more serious complications, such as abscess formation or rupture [13]. 

Dae-Hyuk Heo et al. summarized 20 cases of splenic infarction with acute infectious mononucleosis due to EBV in the medical literature [14]. There are three more cases with the same findings. All these 23 cases have been published between 1961 and 2017 [14]. However, our patient is in a younger age group and previously healthy, who was taking OCPs. She also had a negative monospot test, but EBV was confirmed later on, along with splenic infarcts with splenomegaly and a good clinical outcome. 

In patients with EBV positivity, who are also taking OCPs, the risk of developing splenic infarction may be amplified due to the combined effects of OCP-induced thrombophilia and EBV-induced endothelial dysfunction or splenic congestion [5-8,15]. Clinicians should be aware of this potential interaction when managing patients with EBV infection, particularly those on OCPs, and consider appropriate prophylactic or therapeutic measures to reduce thromboembolic risk.

The management of splenic infarction in IM is typically conservative, focusing on symptomatic relief and the prevention of complications [16]. This patient responded well to supportive care, with improved liver function tests, jaundice resolution, and fatigue recovery. Avoidance of physical exertion and contact sports was crucial in preventing splenic rupture, a potentially life-threatening complication [16]. 

In this case, the diagnosis was facilitated by a combination of clinical presentation, laboratory tests, and imaging studies. The management was largely supportive, focusing on preventing further complications such as splenic rupture. This case underscores the need for clinicians to consider splenic infarction as a potential complication of EBV infection, particularly in patients presenting with severe abdominal pain and evidence of splenomegaly. Early diagnosis and conservative management can lead to favorable outcomes and prevent further morbidity. 

## Conclusions

In conclusion, infectious mononucleosis due to EBV infection is very common in young adults; however, splenic infarction is a rare complication. Our case showed that the patient presented initially with a sore throat followed by abdominal pain and jaundice with a negative monospot test, positive EBV antibodies, and splenic infarctions developing infectious mononucleosis. This suggests that IM should be considered as a differential diagnosis of splenic infarction in young adults, especially in patients taking OCPs. No specific therapy may be needed for splenic infarctions in IM. However, clinicians should be aware of this heightened risk in patients with IM who are using OCPs, and consider strategies to mitigate potential complications, including careful monitoring for signs of splenic involvement and, when appropriate, reassessment of contraceptive options during acute illness. Prompt recognition and management of splenic infarction in these patients are crucial to prevent further morbidity. 

## References

[1] Ebell MH, Call M, Shinholser J, Gardner J (2016). Does this patient have infectious mononucleosis?: the rational clinical examination systematic review. JAMA.

[2] Leung AK, Lam JM, Barankin B (2024). Infectious mononucleosis: an updated review. Curr Pediatr Rev.

[3] Balfour HH Jr, Dunmire SK, Hogquist KA (2015). Infectious mononucleosis. Clin Transl Immunology.

[4] Jenson HB (2000). Acute complications of Epstein-Barr virus infectious mononucleosis. Curr Opin Pediatr.

[5] Antopolsky M, Hiller N, Salameh S, Goldshtein B, Stalnikowicz R (2009). Splenic infarction: 10 years of experience. Am J Emerg Med.

[6] van Hal S, Senanayake S, Hardiman R (2005). Splenic infarction due to transient antiphospholipid antibodies induced by acute Epstein-Barr virus infection. J Clin Virol.

[7] Naviglio S, Abate MV, Chinello M, Ventura A (2016). Splenic infarction in acute infectious mononucleosis. J Emerg Med.

[8] Gang MH, Kim JY (2013). Splenic infarction in a child with primary Epstein-Barr virus infection. Pediatr Int.

[9] Gavriilaki E, Sabanis N, Paschou E, Grigoriadis S, Mainou M, Gaitanaki A, Skargani-Koraka M (2013). Splenic infarction as a rare complication of infectious mononucleosis due to Epstein-Barr virus infection in a patient with no significant comorbidity: case report and review of the literature. Scand J Infect Dis.

[10] Li Y, George A, Arnaout S, Wang JP, Abraham GM (2018). Splenic infarction: an under-recognized complication of infectious mononucleosis?. Open Forum Infect Dis.

[11] Jaroch MT, Broughan TA, Hermann RE (1986). The natural history of splenic infarction. Surgery.

[12] Mamo G, Erickson S, Komanduri K, Zayas D, Aggarwal N (2023). Infectious mononucleosis-induced splenic infarction: perhaps more common in healthy individuals than previously thought. Cureus.

[13] Stuempfig ND, Seroy J (2024 Jan-). Monospot Test. StatPearls [Internet].

[14] Heo DH, Baek DY, Oh SM, Hwang JH, Lee CS, Hwang JH (2017). Splenic infarction associated with acute infectious mononucleosis due to Epstein-Barr virus infection. J Med Virol.

[15] Abdallah AO, Kaur V, Mahmoud F, Motwani P (2017). Image diagnosis: splenic infarction associated with oral contraceptive pills in a healthy young woman. Perm J.

[16] Noor M, Sadough M, Chan S, Singh G (2017). Splenic infarct in a patient with infectious mononucleosis: a rare presentation. J Community Hosp Intern Med Perspect.

